# Ceramide Ehux-C22 Targets the miR-199a-3p/mTOR Signaling Pathway to Regulate Melanosomal Autophagy in Mouse B16 Cells

**DOI:** 10.3390/ijms25158061

**Published:** 2024-07-24

**Authors:** Jiyue Wan, Shumiao Zhang, Guiling Li, Shiying Huang, Jian Li, Zhengxiao Zhang, Jingwen Liu

**Affiliations:** College of Ocean Food and Biological Engineering, Jimei University, Xiamen 361021, China

**Keywords:** Ehux-C22, miR-199a-3p, mouse B16 melanoma cells, melanosomal autophagy, mTOR-ULK1 signaling pathway

## Abstract

Melanosomes are specialized membrane-bound organelles where melanin is synthesized and stored. The levels of melanin can be effectively reduced by inhibiting melanin synthesis or promoting melanosome degradation via autophagy. Ceramide, a key component in the metabolism of sphingolipids, is crucial for preserving the skin barrier, keeping it hydrated, and warding off the signs of aging. Our preliminary study indicated that a long-chain C22-ceramide compound (Ehux-C22) isolated from the marine microalga *Emiliania huxleyi*, reduced melanin levels via melanosomal autophagy in B16 cells. Recently, microRNAs (miRNAs) were shown to act as melanogenesis-regulating molecules in melanocytes. However, whether the ceramide Ehux-C22 can induce melanosome autophagy at the post-transcriptional level, and which potential autophagy-dependent mechanisms are involved, remains unknown. Here, miR-199a-3p was screened and identified as a novel upregulated miRNA in Ehux-C22-treated B16 cells. An in vitro high melanin expression model in cultured mouse melanoma cells (B16 cells) was established by using 0.2 μM alpha-melanocyte-stimulating hormone(α-MSH) and used for subsequent analyses. miR-199a-3p overexpression significantly enhanced melanin degradation, as indicated by a reduction in the melanin level and an increase in melanosome autophagy. Further investigation demonstrated that in B16 cells, Ehux-C22 activated miR-199a-3p and inhibited mammalian target of rapamycin(mTOR) level, thus activating the mTOR-ULK1 signaling pathway by promoting the expression of unc-51-like autophagy activating kinase 1 (ULK1), B-cell lymphoma-2 (Bcl-2), Beclin-1, autophagy-related gene 5 (ATG5), and microtubule-associated protein light chain 3 (LC3-II) and degrading p62. Therefore, the roles of Ehux-C22-regulated miR-199a-3p and the mTOR pathway in melanosomal autophagy were elucidated. This research may provide novel perspectives on the post-translational regulation of melanin metabolism, which involves the coordinated control of melanosomes.

## 1. Introduction

Melanins, a class of natural pigments, maintain the relative stability of skin pigmentation and hair color in mammals. Melanin is synthesized in a special lysosome-associated organelle, i.e., the melanosome, of melanocytes. Skin is protected from UV radiation by maintaining melanin at normal physiological levels. Nevertheless, an abnormal deposition of melanin can lead to different pigmentation disorders, such as chloasma, ephelides, and lentigines [1]. Melanoma is a malignant tumor derived from the neoplastic transformation of melanocytes and occurs at all anatomic sites where melanocytes can be found. Curcumin has the ability to trigger programmed cell death in melanoma cells via the Fas receptor and caspase-8 pathway, regardless of p53 status [2]. Melanoma cells harboring mutant p53 exhibited significant resistance to standard chemotherapy treatments. Consequently, curcumin might overcome this chemoresistance, presenting new potential treatment strategies. Several studies have demonstrated that chalcone derivatives, along with the marine polyphenolic compound known as diphlorethohydroxycarmalol (DPHC), were capable of inhibiting melanin synthesis by downregulating both tyrosinase and microphthalmia-associated transcription factor (MITF) expression [3,4,5]. Additionally, several studies have highlighted the role of autophagy-mediated melanosome degradation in regulating melanocyte biology and physiological skin pigmentation [6,7,8,9]. As an example, treating human keratinocytes and melanocytes with the synthetic autophagy inducer PTPD-12 triggered the autophagic flux, resulting in a reduction in melanin levels [6]. The marliolide derivative, DMF02, was found to effectively reduce melanin levels by promoting melanosome degradation in both melanocytes and keratinocytes [10]. Autophagy involves a series of precise regulatory mechanisms that mediate the sequestration, breakdown, and re-circulation of cellular components. Among the various signaling pathways involved in the activation of autophagy, one of the most well defined is the mammalian target of rapamycin (mTOR) pathway. Kim et al. [6] first discovered that PTPD-12-mediated autophagy-related melanosomal degradation was controlled by ULK1-mediated Beclin-1 phosphorylation in melanocytes. Although autophagy has been well documented in regulating melanin degradation, the precise molecular processes are not fully understood.

Sphingolipids, as bioactive lipid messengers, play essential roles in the control of cell proliferation, inflammation, tumor metastasis, apoptosis, and autophagy [11]. Ceramide, mainly distributed on the epidermis, serves as the primary lipid constituent of the skin and is essential for skin barrier maintenance and moisturization [12]. Similarly, a study by Nugroho et al. highlighted that moisturizers containing ceramide improve SCORing Atopic Dermatitis (SCORAD) scores and trans-epidermal water loss (TEWL) [13]. Additionally, several studies have reported the potential effects of ceramide analogs (C2-ceramide and ceramide PC102) or plant-derived ceramides on melanin synthesis [14,15,16]. A long-chain ceramide compound, C22-ceramide (Ehux-C22), was first isolated from the marine microalga *Emiliania huxleyi* in our laboratory. A preliminary study showed that Ehux-C22 can reduce melanin levels via melanosomal autophagy in B16 cells.

MicroRNAs (miRNAs) play a critical function in regulating gene expression by affecting the translation or degradation of messenger RNAs. These small noncoding RNAs, typically between 19 and 25 nucleotides in size, have been shown to play important roles in a variety of cellular processes [17]. Upon investigating the roles of miRNAs in regulating melanin levels, accumulating evidence has shown that miRNAs may affect melanin metabolism by directly affecting the activity and/or expression of melanin biosynthesis genes to modulate the pigmentation process [18]. For example, miR-125b-5p targeted and reduced MITF gene expression, thereby reducing hyperpigmentation [19]. miR-125b has been shown to regulate the pigmentation gene SH3BP4 [20]. miR-340 targeted Ras homolog family member A (RhoA), ameliorating hyperpigmentation by preventing melanosome transport [21]. Moreover, an increasing amount of research has found that miRNAs play a vital role in the processes of melanosome degradation [22,23,24,25].

miR-199a-3p, a member of the miR-199a gene family, is located on human chromosome 19 from position 10,817,426 to 10,817,496 within intron 16 of the DNM2 gene [26]. It is differentially expressed in various tumor tissues and cells. For instance, in MT-1 cells, overexpression of miR-199a-3p suppressed caveolin-2, leading to enhanced breast cancer cell proliferation and viability [27]. Additionally, an over-expression of miR-199a-3p targeted and inhibited the production of CD44, a type I transmembrane glycoprotein molecule, thereby inhibiting the proliferation of liver cancer cells [28]. Moreover, miR-199a derived from human amniotic membrane stem cell exosomes targeted and inhibited the expression of mTOR, inducing melanosome autophagy degradation in B16F10 cells and thereby reducing cellular melanin levels [22]. However, the precise regulatory roles of miR-199a-3p in melanosomal autophagy remain to be elucidated.

Despite numerous studies showing that miRNAs participate in the control of animal fur pigmentation and melanin production, the precise mechanism by which Ehux-C22-mediated miRNAs modulate melanosome degradation is not fully understood. Here, miR-199a-3p was identified as a novel overexpressed miRNA in B16 cells treated with Ehux-C22. Furthermore, miR-199a-3p could target the mammalian target of rapamycin (mTOR) gene, a key regulatory protein of autophagy. This study offers insights into the influence of the miR-199a-3p/mTOR axis and the signaling pathway on melanosome autophagy in B16 cells. These findings might shed light on the molecular processes behind melanin metabolism, as well as the epigenetic and hereditary mechanisms that regulate hyperpigmentation.

## 2. Results

### 2.1. Screening and Authentication of miRNAs and Autophagy-Associated Target Genes

**Figure 1:**
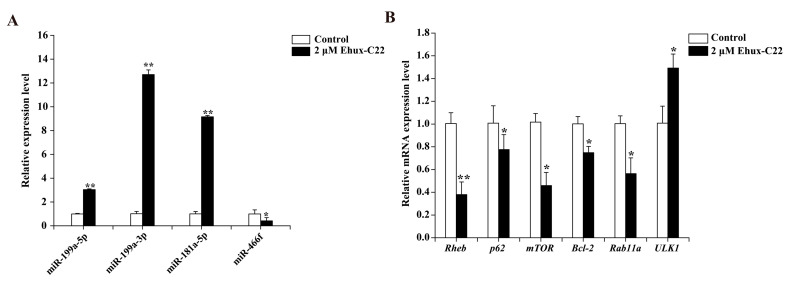
Impact of Ehux-C22 on the regulation of autophagy-associated miRNAs and their respective target genes in B16 cells: (**A**) Analysis of miRNA expression levels; (**B**) Assessment of target gene mRNA levels. Error bars indicate means ± SD of three biological replicates. * *p* < 0.05 and ** *p* < 0.01.

As shown in Figure 1A, treatment with 2 µM Ehux-C22 resulted in a significant increase in the expression of miR-199a-5p, miR-199a-3p, and miR-181a-5p in B16 cells (*p* < 0.01). Conversely, the expression of their target genes, such as Ras homolog enriched in brain (*Rheb*), ubiquitin-binding protein (p62), mammalian target of rapamycin (*mTOR*), B-cell lymphoma-2 (*Bcl-2*), and RAS oncogene family member 11a (*Rab11a*), were significantly downregulated (*p* < 0.05) (Figure 1B). The miR-466f was markedly decreased (Figure 1A), while the expression level of its target gene unc-51-like kinase 1 (*ULK1*), significantly increased (*p* < 0.05) (Figure 1B). Although miR-30a-5p is predicted to target genes such as *Beclin-1* and *Sirtuin 1*, the expression levels of these genes did not exhibit a negative correlation with miR-30a-5p expression. Among the five differentially expressed miRNAs, miR-199a-3p exhibited a relatively high abundance. Thus, miR-199a-3p and mTOR were selected for subsequent analyses.

### 2.2. Analysis of the Targeted Relationship between miR-199a-3p and Its Target Gene mTOR

**Figure 2:**
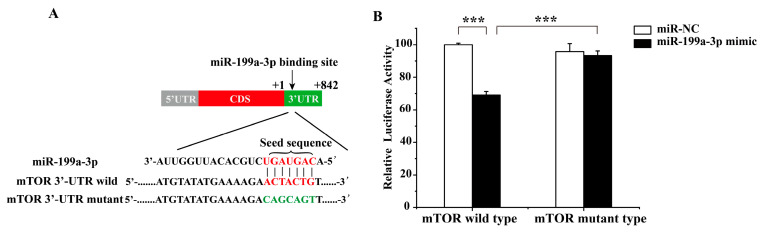
The targeting relationship between miR-199a-3p and the target gene mTOR: (**A**) The miR-199a-3p target locus was within the 3′UTR of mTOR. Red represented the seed sequence, and green represented the mutant region. (**B**) The dual-luciferase reporter assay was employed to measure the Luciferase relative activity. Error bars indicate means ± SD of three biological replicates. *** *p* < 0.001.

A schematic diagram illustrating the binding site sequence and mutation sequence of miR-199a-3p with the 3′UTR of *mTOR* is presented in Figure 2A. The luciferase reporter gene assay results are shown in Figure 2B. The relative luciferase level in the cells transfected with the wild-type vector containing miR-199a-3p and mTOR was remarkably reduced compared to the other experimental groups, indicating that mTOR is a target gene of miR-199a-3p (Figure 2B).

### 2.3. miR-199a-3p Expression Level

**Figure 3:**
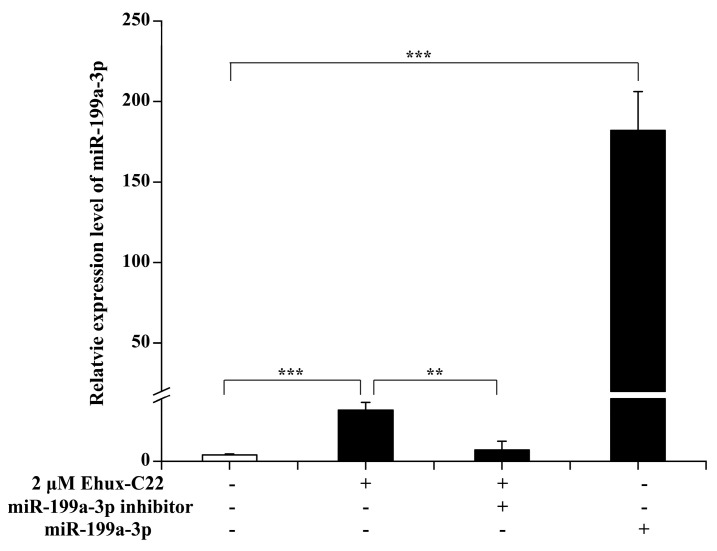
The expression level of miR-199a-3p in B16 cells. After transfection with miR-199a-3p or miR-199a-3p inhibitor for 6 h, B16 cells were incubated in DMEM with 0.2 µM α-MSH, and Ehux-C22 was added according to the group, followed by incubation for 48 h. Error bars indicate means ± SD of three biological replicates. ** *p* < 0.01; *** *p* < 0.001.

The expression levels of miR-199a-3p were analyzed in different groups (Figure 3). Compared to the control, 2 µM Ehux-C22 significantly increased the expression of miR-199a-3p (*p* < 0.001). Moreover, transfection with miR-199a-3p led to a significant upregulation of miR-199a-3p (*p* < 0.001). Conversely, a significant reduction in miR-199a-3p expression was seen with simultaneous exposure of cells to Ehux-C22 and miR-199a-3p inhibitor in comparison to the groups treated with just 2 µM Ehux-C22 (*p* < 0.01). These results indicated that Ehux-C22 significantly promoted the expression of miR-199a-3p.

### 2.4. Effects of miR-199a-3p on Melanin Levels in B16 Cells

**Figure 4:**
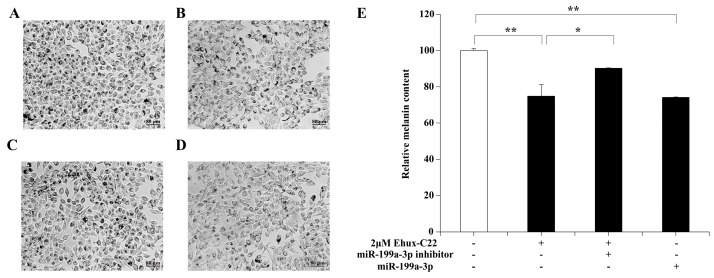
Effect of miR-199a-3p on melanin levels in B16 cells: (**A**–**D**) Images were observed by optical microscopy at 100× magnification. (**A**) Blank control: 0.2 µM α-MSH; (**B**) 0.2 µM α-MSH+2 µM Ehux-C22; (**C**) 0.2 µM α-MSH+2 µM Ehux-C22 + miR-199a-3p inhibitor; (**D**) 0.2 µM α-MSH+miR-199a-3p. (**E**) The absorbance (490 nm) was measured to calculate the melanin level. Error bars indicate means ± SD of three biological replicates. * *p* < 0.05; ** *p* < 0.01.

An in vitro high melanin expression model in cultured mouse melanoma cells (B16 cells) was established by using 0.2 μM alpha-melanocyte-stimulating hormone(α-MSH) and was subsequently analyzed (Figure S1). To confirm whether miR-199a-3p mediated the pigmentation effect of Ehux-C22, B16 cells stimulated with α-MSH were treated with either Ehux-C22 or miR-199a-3p, and the melanin levels were subsequently analyzed. A 2 µM Ehux-C22 treatment notably inhibited the increase in intracellular melanin levels induced by α-MSH (*p* < 0.01) (Figure 4). The treatment of B16 cells with miR-199a-3p exhibited similar effects. However, when cells were cotreated with Ehux-C22 and miR-199a-3p inhibitor, the inhibitory effect on melanin levels decreased. The above results indicated that Ehux-C22 could suppress the production of melanin in B16 cells by upregulating miR-199a-3p expression.

### 2.5. The Effect of miR-199a-3p on Melanosome Autophagy in B16 Cells

**Figure 5:**
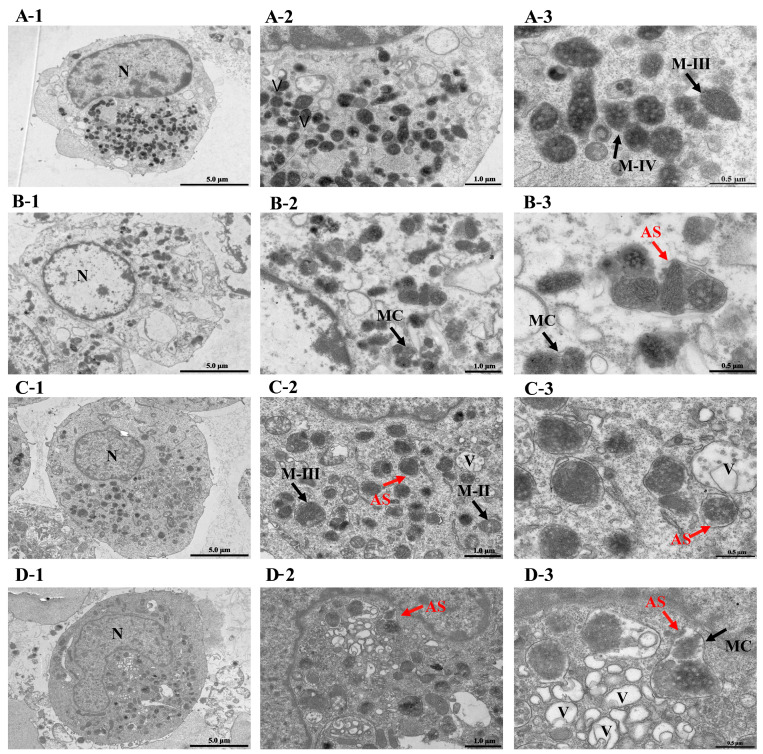
Electron micrograph of melanosomes structure in B16 cells: (**A**) Blank control: 0.2 µM α-MSH; (**B**) 0.2 µM α-MSH+2 µM Ehux-C22; (**C**) 0.2 µM α-MSH + 2 µM Ehux-C22 + miR-199a-3p inhibitor; (**D**) 0.2 µM α-MSH+miR-199a-3p. The scale bars represent 5.0 µm (**A-1**–**D-1**), 1.0 µm (**A-2**–**D-2**) and 0.5 µm (**A-3**–**D-3**). M-I/II/III/IV: I/II/III/IV stage melanosome; V: vacuole; AS: autolysosome; MC: melanosome complex; N: nucleus.

TEM analysis revealed the degradation of melanosomes in B16 cells subjected to 2 µM Ehux-C22 treatment or miR-199a-3p/inhibitor transfection (Figure 5). Melanosome complexes were engulfed by melanoma cell lysosomes in B16 cells after Ehux-C22 treatment. In particular, the complexes were surrounded by an autophagic lysosome bilayer membrane structure, and degraded melanin particles were present within the autophagic lysosomes (Figure 5B-3). However, the addition of the miR-199a-3p inhibitor reversed the melanosome autophagy induced by Ehux-C22 (Figure 5C-3). Moreover, there were many autophagolysosomes present in the miR-199a-3p-transfected group (Figure 5D-3).

**Figure 6:**
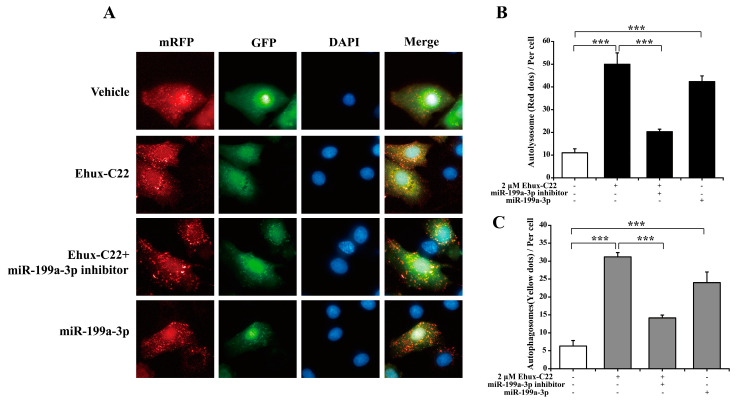
Impact of miR-199a-3p on autophagic flux in B16 cells: (**A**) Two hours after transfection with mRFP-GFP-LC3 adenovirus, B16 cells were treated with control: 0.2 µM α-MSH, 0.2 µM α-MSH+2 µM Ehux-C22, 0.2 µM α-MSH+miR-199a-3p or 0.2 µM α-MSH+Ehux-C22+miR-199a-3p inhibitor. After 36 h, fluorescent LC3-positive puncta were observed by confocal microscopy. (**B**) Autolysosomes (red puncta) were quantified as the number of puncta (red puncta/total puncta) in the merged images. (**C**) Autophagosomes (yellow puncta) were quantified as the number of puncta (yellow puncta/total puncta) in the merged images. *** *p* < 0.001.

Furthermore, we measured the autophagic flux of B16 melanoma cells by mRFP-GFP-LC3 fluorescence microscopy. The activation of autophagy in B16 cells by 2 µM Ehux-C22 treatment and miR-199a-3p transfection was further confirmed by significant autolysosome formation and the accumulation of GFP-LC3 puncta in response to Ehux-C22 treatment or miR-199a-3p after LC3-GFP-mRFP transfection (red puncta; Figure 6A, *p* < 0.001). However, when a miR-199a-3p inhibitor was added, the autophagy flux induced by Ehux-C22 was reversed. These results suggested that treatment with 2 µM Ehux-C22 triggers the formation of autophagosomes via miR-199a-3p, promotes autophagic flux, and induces autophagy. In comparison to the vehicle group, 2 µM Ehux-C22 treatment increased the formation both of mRFP-GFP-LC3 red puncta (autolysosomes) (Figure 6B) and yellow puncta (Figure 6C). Similar results were present in the miR-199a-3p treatment group (Figure 6B,C). Moreover, when the miR-199a-3p inhibitor was added, mRFP-GFP-LC3 red puncta (autolysosomes) formation decreased compared with that in the 2 µM Ehux-C22 group (Figure 6B), and the numbers of yellow puncta (autophagosomes) decreased (Figure 6C).

### 2.6. miR-199a-3p Regulates the Expression Levels of mTOR

**Figure 7:**
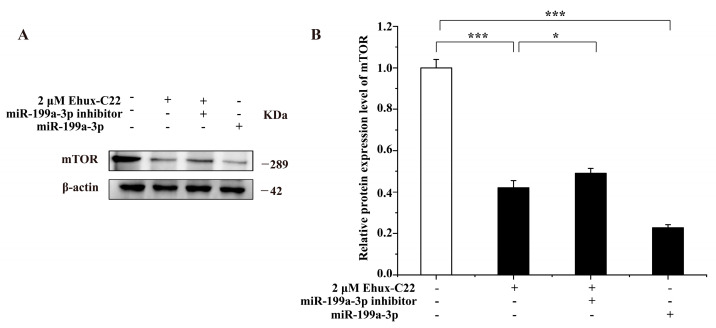
Impact of miR-199a-3p on the expression of mTOR in B16 cells: (**A**) Representative band from a Western blot for mTOR; (**B**) quantitative analysis by ImageJ 1.54d 30. * *p* < 0.05; *** *p* < 0.001.

Ehux-C22 treatment or overexpression of miR-199a-3p suppressed mTOR expression, which was partially reversed by miR-199a-3p inhibitor (Figure 7).

### 2.7. Effect of miR-199a-3p on the Expression Levels of Autophagy-Related Factors in the mTOR-ULK1 Signaling Pathway

**Figure 8:**
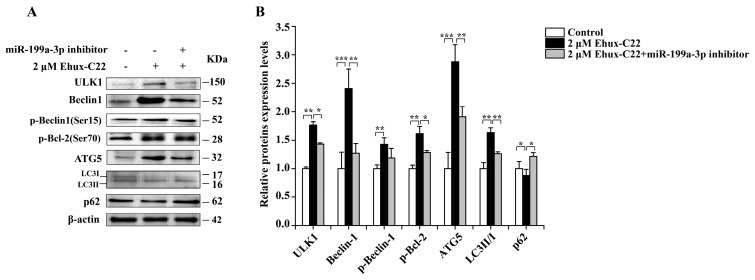
Protein expression levels of key autophagy-related components of the mTOR-ULK1 signaling pathway: (**A**) Representative Western blot bands for unc-51-like autophagy activating kinase 1 (ULK1), Beclin-1, p-Beclin-1 (Ser15), p-Bcl-2 (Ser70), autophagy-related gene 5 (ATG5), p62, and microtubule-associated protein light chain 3 (LC3-II/I). (**B**) Relative quantitative analysis of proteins by ImageJ 1.54d 30. * *p* < 0.05; ** *p* < 0.01; *** *p* < 0.001.

Treatment with 2 µM Ehux-C22 significantly downregulated mTOR expression (*p* < 0.001) and upregulated the protein expression of its direct target unc-51-like autophagy activating kinase 1 (ULK1) (*p* < 0.01) to promote the upregulation of phosphorylated Beclin-1 (Ser15) expression (*p* < 0.01) (Figure 8). Moreover, enhanced phosphorylation of B-cell lymphoma-2 (Bcl-2) at Ser70 (*p* < 0.01) triggered the dissociation of the Beclin-1/Bcl-2 complex and the activation of melanosomal autophagy. Additionally, the enhanced expression of p-Beclin-1 facilitated the process of melanosomal autophagy by increasing the levels of the autophagy-associated proteins autophagy-related gene 5 (ATG5) and microtubule-associated protein light chain 3 (LC3-II/I) (*p* < 0.01), leading to the extension of autophagic membranes. Additionally, we observed a decrease in polyubiquitin-binding protein p62 (*p* < 0.05), which was degraded by autophagy, in B16 cells treated with 2 µM Ehux-C22. In contrast, cotreatment of B16 cells with 2 µM Ehux-C22 and miR-199a-3p inhibitor increased mTOR expression levels (*p* < 0.05), resulting in a reversal of the expression of downstream autophagic cascade-related proteins. These findings suggest that Ehux-C22-induced melanosome autophagy inhibits melanin levels by inactivating mTOR-ULK1 signaling.

## 3. Discussion

Ceramides are fundamental components of skin lipid composition, contributing to the structural integrity and maintaining the water retention function of the skin [29,30]. Leo et al. suggested that rice ceramide supplementation effectively improved skin barrier function, and reduced wrinkle severity and pigmentation [12]. The changes in the ceramide content of the skin are closely associated with a number of skin diseases, such as atopic dermatitis [31]. With an in-depth understanding of the function of ceramides, research on ceramide regulation of melanin levels has attracted significant attention. Exogenous C2-ceramide [32] and the synthetic ceramide analog PC102 [15] have been shown to promote decoloration by reducing the expression levels of melanin biosynthesis-associated enzymes or genes, such as MITF, tyrosinase (TYR), and tyrosinase-related protein (TYRP). Although ceramides have been shown to play roles in various epigenetic controls, such as miRNA expression, DNA methylation, and histone modification [33], whether the regulation of ceramides on melanin levels involves similar epigenetic mechanisms remains uncertain. In recent studies, miRNAs have emerged as crucial regulators of melanin levels. Ehux-C22 induced miR-199-3p overexpression and triggered melanosomal autophagy. Dual-luciferase reporter tests validated the targeting interaction between miR-199a-3p and mTOR (Figure 2). Several reports have demonstrated that miRNAs play a crucial role in regulating melanin biosynthesis. For instance, endogenous miR-508-3p in alpaca melanocytes was shown to directly regulate the MITF expression level targeting the 3′UTR of MITF, and the overexpression of miR-508-3p regulated the expression of MITF, leading to a reduction in the expression of key melanin genes, such as TYR and TYRP-2 [34]. Furthermore, it has been reported that miRNAs regulate melanin levels through the modulation of autophagy-associated gene expression. For example, Polygonatum odoratum lectin (POL) induced autophagy in A375 human melanoma cells by suppressing miR-1290 levels and enhancing Beclin-1 expression [35]. Treatment with licochalcone A induced the expression of miR-142-3p in human malignant melanoma A375 cells and mouse B16 cells. Upregulated miR-142-3p expression has been shown to inhibit the expression of the target gene Ras homolog enriched in brain (Rheb), leading to the induction of autophagy targeting mTOR and resulting in a reduction in melanin levels [24]. Exosomal miR-199a, derived from human amniotic stem cells, has been shown to act on B16 cells to reduce melanin levels by inhibiting mTOR expression, ultimately leading to melanosome degradation [22].

The complex regulatory mechanism of melanin metabolism is involved in various factors. Recently, it was suggested that a network of mRNAs and miRNAs plays a role in the maturation and trafficking of melanosomes [36]. In addition, miR-342-5p has been shown to target melanophilin(MLPH) to inhibit melanosome transport and induce melanosome aggregation [37]. Here, we found that Ehux-C22-induced differentially expressed miRNAs were significantly enriched in the autophagy pathway in B16 cells (Figures S2 and S3), and were predicted to target genes related to the mTOR signaling pathway and participate in the autophagic process of melanosomes (Figure S4). Taken together, the differentially expressed miRNAs induced by Ehux-C22 may target genes related to the mTOR signaling pathway involved in melanosome autophagy in B16 cells. Ehux-C22 treatment induced the overexpression of miR-199a-3p in B16 cells, leading to an increase in acid phosphatase activity (Figure S5) and a decrease in tyrosinase activity (Figure S6). In the process of melanosomal autophagy, melanosomes are sequestered and create autophagosomes, and then fused with lysosomes to form autophagolysosomes (Figure 6), in which the degradation of melanosomes are initiated due to the acidic environment inside the lysosome. Lysosomes contain plenty of acid hydrolases that can digest different kinds of biological molecules, and acid phosphatase is the hallmark enzyme of lysosomes. As a result, acid phosphatase activity greatly increased. tyrosinase and tyrosinase-related proteins are mainly distributed on the melanosome membrane and these enzymes exhibit high catalytic activity under neutral pH conditions. In melanosome autophagy, the lower pH value (4.0–5.0) in the autophagolysosome inhibited the activity of tyrosinase. Autophagy is an intracellular degradation process in which components of the cell are encapsulated in a double-membrane structure to form autophagosomes, which subsequently fuse with lysosomes, where acid hydrolases break down material within the autophagosomes. Acid phosphatase in lysosomes plays a key role in maintaining the acidic environment inside lysosomes, which is necessary for the activity of hydrolases [38,39]. In fact, during the process of autophagy, the acidification of lysosomes and the enhancement of enzyme activity are crucial for the degradation of substances within the autophagosome. For example, recent research showed that a natural nucleoside analog, cordycepin, has the ability to significantly improve lysosomal acid phosphatase activity and enhance the activity of the lysosomal representative protease cathepsin B (CTSB), thereby promoting lysosomal maturation enhanced autophagy and decreased cell senescence level [40]. Again, a study by Tai et al. demonstrated that the activity of lysosomal acid phosphatase decreased in senescent cells, leading to the impairment of autophagy [41]. Lysosomes maintain their pH gradient through proton pump V-ATPase, while autophagosome–lysosome fusion permits the acidic environment of lysosomes to come into contact with substances within the autophagosome, thereby facilitating the degradation process [42]. Therefore, there is a close relationship between autophagy and acid phosphatase in lysosomes. Lysosomes provide the site for autophagic degradation, while acid phosphatase ensures the appropriateness of the lysosomal internal environment, and the two work together to complete intracellular material cycling and quality control. In this study, the overexpression of miR-199a-3p promoted melanosome degradation and autophagic flux in B16 cells, indicating that miR-199a-3p reduced melanin levels in B16 cells by inducing melanosome autophagy (Figure 5 and Figure 6).

Autophagy is regulated by several pathways, including the adenosine 5’-monophosphate (AMP)-activated protein kinase (AMPK) signaling pathway, the phosphoinositide 3-kinase (PI3K) pathway, and mitogen-activated protein kinase (MAPK) and mTOR signaling pathways [43,44,45]. Several compounds, such as PTPD-12, licochalcone A, and ISL, regulated melanosomal autophagy in human epidermal keratinocytes, B16 cells, and HaCaT cells via the PI3K/AKT/mTOR signaling pathways [6,24,46]. Previous research indicated that 2 μM Ehux-C22 induced the autophagy of melanosomes in mouse B16 cells by activating proteins in the c-Jun N-terminal kinase/oncogene protein (JNK/c-Jun) signaling pathway, such as p-JNK at Thr183/Tyr185, p-c-Jun at Ser63, p-Bcl-2 at Ser70, and p-Beclin-1 at Ser15. In this study, 2 µM Ehux-C22 significantly inhibited the expression of Rheb, leading to a decrease in the expression of the downstream target mTOR (Figure 7 and Figure 8). ULK1 is a direct downstream target of mTOR, and the inhibition of mTOR expression activated ULK1 to initiate autophagosome formation, thereby initiating melanosome autophagy in B16 cells. Therefore, the induction of melanosome autophagy by Ehux-C22 might involve synergistic regulation through multiple signaling pathways. The JNK-c-Jun and mTOR-ULK1 pathways communicate with each other through interactions with Beclin-1, triggering the phosphorylation of Beclin-1 at Ser15, which results in the formation of III PI3K complex (ATG14/Beclin-1/Vsp34) and promotes melanosome autophagy. The amount of Bcl-2 attached to Beclin-1 within the cell determines the level of autophagy to a certain extent, while decreased binding of Beclin-1 and Bcl-2 initiated cellular autophagy [47]. The release of Beclin-1, an essential autophagy modulator, promotes autophagy. Similarly, the autophagy inducer PTPD-12 promotes the formation of early autophagosomes in human melanocytes and induces the translocation of melanosomes to autophagosomes by activating ULK1/p-Beclin-1 (Ser15) [6]. In breast cancer cells, diosgenin, a traditional Chinese medicine monomer, has been proven to inhibit Akt and mTOR phosphorylation while enhancing JNK phosphorylation, leading to the inhibition of cell proliferation and the induction of apoptosis [48]. In T24 cells, pentacyclic triterpene-amino-acid induced autophagy by activating the JNK pathway and inhibiting the PI3K/AKT/mTOR pathway [49]. In fact, the interaction between multiple protein nodes in the mTOR and JNK signaling pathways determines the ultimate fate of cells to a certain extent due to the complex network regulatory effects formed by these two signaling pathways [50,51].

**Figure 9:**
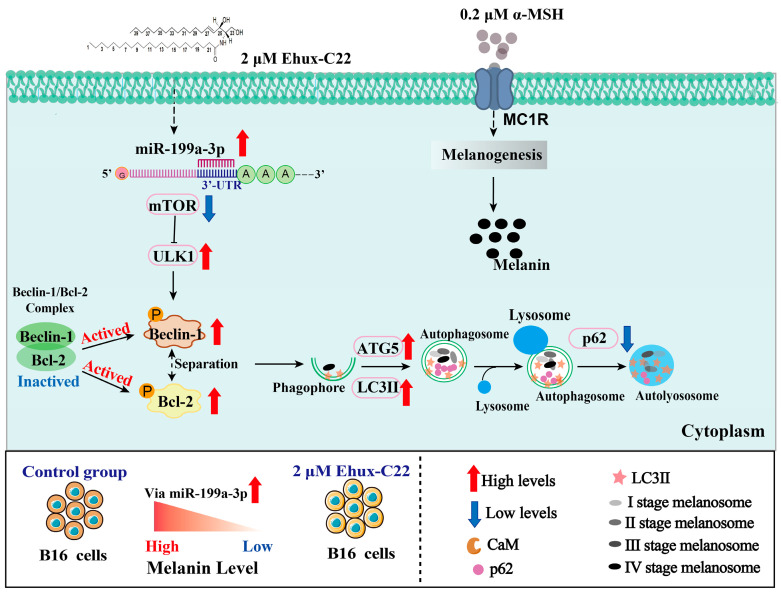
Signaling pathway through which Ehux-C22 regulates miR-199a-3p targeting mTOR to induce melanosome autophagy in B16 cells.

As expected, mTOR protein expression levels recovered significantly in the miR-199a-3p inhibitor and Ehux-C22 co-treated group (Figure 7). This result suggested that in addition to inducing melanosome autophagy directly through the mTOR-ULK1 signaling pathway, Ehux-C22 regulated melanosome autophagy in B16 cells by modulating the expression of miR-199a-3p. This study revealed that miR-199a-3p played a role in Ehux-C22-induced melanosome autophagy in B16 cells by targeting mTOR to activate important components of the mTOR-ULK1 signaling pathway (Figure 9). Various miRNAs play important roles in regulating melanosome autophagy in different types of cells. For instance, miR-199a derived from human amniotic mesenchymal stem cell exosomes has been shown to inhibit pigmentation, directly block the expression of mTOR, and induce melanosomal autophagy in B16F10 cells [22]. Additionally, the inhibition of miR-1299 in human keratinocytes leads to increased expression of its target gene arginase-2 (AGR2) by activating the AMPK/mTOR signaling pathway, ultimately promoting pigmentation [23]. These results demonstrate that melanin degradation involves complex posttranscriptional regulation.

The same miRNA regulates a variety of metabolic processes by targeting different signaling pathways. For instance, overexpression of miR-199a-3p suppressed the proliferation and growth of melanoma cells, prompting cell apoptosis [52]. While, in gastric carcinoma cells, miR-199a-3p targeted zinc fingers and homeoboxes 1 (ZHX1), resulting in the proliferation of gastric tumor cells and inhibition of apoptosis [53]. Moreover, in osteosarcoma cells, miR-199a-3p targeted mTOR, mesenchymal to epithelial transition factor(Met), signal transducer and activator of transcription 3(Stat3), adenylate kinase 4(AK4), and AXL receptor tyrosine kinase (AXL) respectively, blocking the proliferation, invasion, and metastasis of tumor cells through a complex signaling network pathway [54,55,56]. Here, we found that miR-199a-3p played an important role in the degradation of melanin. Moreover, miRNAs not only regulate autophagy by targeting signaling pathways but also directly target downstream factors of autophagy to regulate autophagy. For example, the downregulation of miR-216b-induced autophagy has been shown to result in drug resistance to vemurafenib in human A375 melanoma cells [57]. In RBL2H3 cells, promoting the expression of miR-135-5p has been shown to downregulate the expression of the target gene p62, thereby increasing the protein levels of LC3II/I and Beclin-1, promoting the formation of autolysosomes, inducing cell autophagy [58]. Lysosomes contain acid hydrolases that break down cellular materials and play a critical part in various forms of autophagy [59]. miRNAs regulated melanosome autophagy by targeting genes encoding lysosomal hydrolases in lysosomes, leading to melanin degradation. For instance, isoimperatorin, which acts on human HaCaT keratinocytes, has been shown to downregulate the expression of miR-3619, resulting in the significant upregulation of lysosomal cysteine protease B (CSTB) and cysteine protease D (CSTD) expression, inducing melanosome autophagy to reduce pigmentation [25]. The present study revealed that Ehux-C22 promoted melanosomal autophagy in B16 cells by modulating the downregulation of miR-466f, which directly targets ULK1 to increase its expression. Conversely, the increase in miR-199a-5p levels directly inhibited p62 expression, and miR-181a-5p inhibited Bcl-2 expression (Figure 1). These miRNAs, along with miR-199a-3p, collectively participate in regulating melanosome autophagy, but further experimental data are needed for validation.

Our results demonstrated that Ehux-C22 regulated melanosome autophagy in B16 cells by modulating the expression of miR-199a-3p, inhibiting mTOR, and activating key molecules of the mTOR-ULK1 signaling pathway. Our results help to elucidate the regulatory mechanism of epigenetic pigmentation and support the development and application of marine microalgal natural products.

## 4. Materials and Methods

### 4.1. Material Sources

Ceramide Ehux-C22 (Cer (d18:1/22:0)) was isolated in our laboratory from *E. huxleyi* BOF92 strain. The B16 melanoma cells were from the Cell Bank of the Chinese Academy of Sciences (Shanghai, China).

### 4.2. Reagents

Chemicals used in this study, unless otherwise specified, were obtained from Sinopharm Chemical Reagent Co. (Shanghai, China). Dulbecco’s modified Eagle’s medium (DMEM), and the penicillin/streptomycin (PS) mixture were all obtained from Thermo Fisher Scientific (Waltham, MA, USA). α-MSH was bought from Sigma. Fetal bovine serum (FBS) was from Gemini (Woodland, CA, USA). Lipofectamine 2000 was acquired from Invitrogen (Carlsbad, CA, USA). The miR-199a-3p vector (miR-199a-3p) was constructed by Sangon Biotech (Shanghai, China). miR-NC, miR-199a-3p mimic, and miR-199a-3p inhibitor were all obtained from Guangzhou RiboBio Co., Ltd. (Guangzhou, China). Dimethyl sulfoxide (DMSO) and 4% paraformaldehyde were bought from Solarbio (Beijing, China). The mRFP-GFP-LC3 adenoviral vectors were acquired from HanBio Technology (Shanghai, China). The WesternBright ECL Western blotting system was obtained from Advansta (Menlo Park, CA, USA).

### 4.3. Cell Culture and Ehux-C22 Treatment

B16 cells were cultured with RPMI 1640 medium provided with 10% FBS and 1% PS (5% CO_2_, 37 °C) overnight. Dissolved in DMSO and MTBE (1:1), 2 µM Ehux-C22 was added to the cells with α-MSH in DMEM.

### 4.4. Construction of the miR-199a-3p Overexpression Vector

The CMV/EGFP expression vector was used for miRNA overexpression. The following PCR primer sequence was used: 5′-CGTGCAGCTCGCCGACCACTA-3′. The sequences of annealing oligonucleotides were as follows:

5′-AATTCGACAGTAGTCTGCACATTGGTTAGTTTTGGCCACTGACTGACTAACCAATGCAGACTACTGTA-3′

5′-CCGGTACAGTAGTCTGCATTGGTTAGTCAGTCAGTGGCCAAAACTAACCAATGTGCAGACTACTGTCG-3′

### 4.5. miRNA Transfection and Cell Sample Collection

B16 cells were cultured until they reached approximately 75% confluence. Subsequently, the miR-199a-3p vector was transfected into the cells using the Lipofectamine 2000, Thermo Fisher Scientific, Waltham, MA, USA. The cells were grouped and treated as follows: 2 µM Ehux-C22; miR-199a-3p inhibitor+2 µM Ehux-C22; and miR-199a-3p. Three biological replicates were used. Six hours post-transfection, the culture medium was replaced with DMEM including α-MSH and/or 2 µM Ehux-C22. Cell samples were harvested 48 h after transfection for subsequent analysis, including melanin levels, tyrosinase activity, acid phosphatase enzyme activity, qRT–PCR, western blot, dual-luciferase reporter assay, autophagic flux, and transmission electron microscopy analysis.

### 4.6. Quantitative Reverse Transcription-Polymerase Chain Reaction (qRT-PCR)

Total RNA was isolated utilizing the TRIzol reagent for RNA isolation as per the manufacturer’s guidelines.

For miRNA, first-strand cDNA was synthesized using the miRcute Plus miRNA First-Strand cDNA Kit, TIANGEN, Beijing, China. qRT–PCR was performed on a Roche Real-time PCR System using a SYBR Green miRcute Plus miRNA qPCR Kit and 96-well plates, TIANGEN, Beijing, China.

For mRNA, first-strand cDNA was synthesized using HiScript III RT SuperMix for qPCR, Vazyme, Nanjing, China. qRT–PCR was performed on a Roche Real-time PCR System using Universal ChamQ Universal SYBR qPCR Master Mix, Vazyme, Nanjing, China and 96-well plates. β-Actin and U6 were used for normalization, and the expression levels of miRNAs and genes were calculated by the 2^−ΔΔCt^ method. The primer sequences for the miRNAs tested can be found in Table S1, and those for the genes tested are listed in Table S2.

### 4.7. Dual-Luciferase Reporter Assay

HepG2 cells were cultured in 500 µL of medium until the cell confluence reached about 70%. Subsequently, cells were co-transfected using Lipofectamine 2000 with 600 µg of a recombinant vector combined with 20 pmol of either miR-199a-3p mimic or miR-NC. Cells that were transfected solely with the recombinant vector served as the control group. Forty-eight hours following transfection, cells were collected and processed to measure relative luciferase activity, following the protocol outlined in the Dual-Luciferase Reporter Kit, Promega, Madison, WI, USA.

### 4.8. Melanin Level Assay

Following the transfection with either miR-199a-3p or a miR-199a-3p inhibitor for a duration of 6 h, B16 cells were subsequently transferred to DMEM medium. This medium also included 0.2 µM α-MSH and/or Ehux-C22, and the cells were cultured for an additional 48 h. After the 48 h incubation, the cells underwent a harvesting process involving trypsinization. The harvested cells were then dissolved in a solution comprising 1 M NaOH and 10% DMSO, maintained at a temperature of 80 °C for one hour. Finally, the absorption value at 490 nm wavelength was calculated using a microplate reader.

### 4.9. Transmission Electron Microscopy (TEM) Analysis

The collected cell samples were treated with 2.5% glutaraldehyde overnight at 25 °C. After exposure to 1% osmium tetroxide for 2 h, the cells were subjected to dehydration through a gradual transition of ethanol and acetone. Subsequently, the cells were embedded in Epon-Araldite resin and stained with 2.6% lead citrate for examination by using a Hitachi TEM microscope, Hitachi High-Tech Corporation, Tokyo, Japan.

### 4.10. Adenoviral Transfection and Measurement of Autophagic Flux

In order to track autophagic flux, B16 cells were cultured to 70% confluence and treated with adenoviral particles at an MOI of 100 in serum-free medium at 37 °C for 2 h. Following 48 h of miRNA transfection or Ehux-C22 treatment, the cells were treated with 4% paraformaldehyde for 30 min, and the nuclei were stained with DAPI solution for 10 min at 25 °C. Subsequently, the cells were analyzed using a confocal microscope. The quantity of RFP and GFP puncta was quantified in 5 different fields, with no less than 50 cells being assessed in each experimental group.

### 4.11. Western Blot

B16 cell samples were harvested 48 h after transfection, and three sets of cell samples from independent cell culture trays were pooled to form one biological replicate for total protein extraction and blotting analysis. Total protein was extracted with lysis buffer and the concentration of protein was measured using a BCA reagent, Thermo Fisher Scientific, Waltham, MA, USA. Then, 40–60 µg protein samples were loaded onto an electrophoresis gel and transferred onto nitrocellulose (NC) membranes. The membranes were then exposed to specific primary antibodies, followed by a secondary antibody conjugated with peroxidase, and the bound proteins were revealed using the WesternBright ECL Western blotting kit, Thermo Fisher Scientific, Waltham, MA, USA. Signals were visualized and quantified relatively with an Azure c400 Imaging System. To ensure equal loading, β-actin was utilized as a protein loading control. The primary antibodies utilized can be found in Table S3. The grayscale of the bands was measured three times to calculate the mean and standard deviation.

### 4.12. Statistical Analysis

Upon testing for heterogeneity of data variances, statistical analyses were conducted using the SPSS 16.0 software (Chicago, IL, USA) via one-way analysis of variance (ANOVA), followed by Duncan’s analysis for multiple comparisons. Quantitative data were given as means ± SD. * *p* < 0.05, ** *p* < 0.01.

## Data Availability

No publicly archived datasets available for this work.

## Supplementary Materials

The following supporting information can be downloaded at: https://www.mdpi.com/article/10.3390/ijms25158061/s1.
