# Supplementary material for: Ceramide Ehux-C22 Targets the miR-199a-3p/mTOR Signaling Pathway to Regulate Melanosomal Autophagy in Mouse B16 Cells
Source: Int J Mol Sci. 2024 Jul 24;25(15):8061. doi: 10.3390/ijms25158061 (PMC11312279; doi:10.3390/ijms25158061)
Supplement: Supplementary file 1 [file ijms-25-08061-s001.zip › ijms-3093259-supplementary.pdf]

Supplementary Materials for Original Paper:

Ceramide Ehux-C22 targets the miR-199a-3p/mTOR signaling pathway to regulate melanosomal autophagy in mouse B16 cells

Journal name: 《*International Journal of Molecular Sciences*》

Jiyue Wan, Shumiao Zhang, Guiling Li, Shiying Huang, Jian Li, Zhengxiao Zhang* Jingwen Liu*

College of Ocean Food and Biological Engineering, Jimei University, Xiamen 361021, P. R. China

*Co-correspondence authors:

Zhengxiao Zhang, Ph.D.

E-mail: zxzhang@jmu.edu.cn

Jingwen Liu, Ph.D.

E-mail: ljwsbch@163.com

Pages: 16

Figures:6

Tables: 3

Supplementary information Appendix

1. Supplementary Materials and Methods

Acid phosphatase activity assay

Tyrosinase Assay

2. Supplementary Figures:

Figure S1. Effect of treatment with α-MSH on melanin level in B16 cells

Figure S2. Gene Ontology annotation of miRNA target genes

Figure S3. KEGG pathway enrichment analysis of miRNA target genes

Figure S4. Ehux-C22-induced miRNAs were involved in the regulation of melanosomal autophagy in B16 cells

Figure S5. Effect of overexpression of miR-199a-3p on acid phosphatase activity treated with α-MSH in B16 cells.

Figure S6. Effect of overexpression of miR-199a-3p on tyrosinase activity after α-MSH-treated in B16 cells.

3. Supplementary Tables:

Table S1. Primer sequences used in qRT-PCR of miRNA.

Table S2. Oligonucleotide primers used for qRT-PCR.

Table S3. List of antibodies used in this study.

1. Supplementary Materials and Methods

Acid phosphatase activity assay

Cells were collected from growth media after transfection with miR-NC or miR-199a-3p and washed twice with PBS. The lysosomes of cells were extracted according to the instructions of lysosome isolationkit (GENMED, USA). The acid phosphatase activity of lysosomes was then tested using an acid phosphatase assay kit (Beyotime,China).

Tyrosinase Assay

In 12-well microplates, B16 cells were added and incubated with miR-NC or miR-199a-3p. The cells were then lysed with PBS containing 1% Triton X-100. Utilizing a BCA Assay Kit, the protein concentration in the lysate was measured. Each well of a 96-well microplate received 90 μL of individual lysates containing an equal amount of protein (adjusted with lysis buffer), followed by 10 μL of 1 mg/mL L-DOPA for 60 min at 37 °C. The absorbance value was determined with a microplate reader at 475 nm.

2.Supplementary Figures:

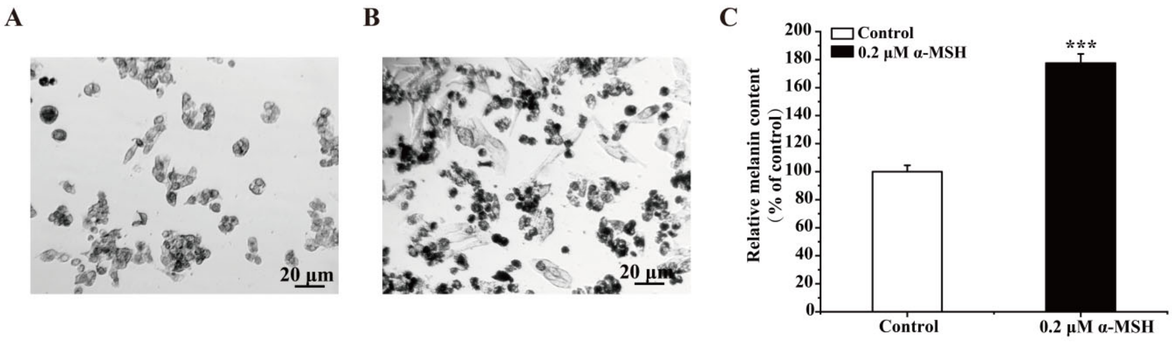

Figure S1. Effect of treatment with α-MSH on melanin level in B16 cells

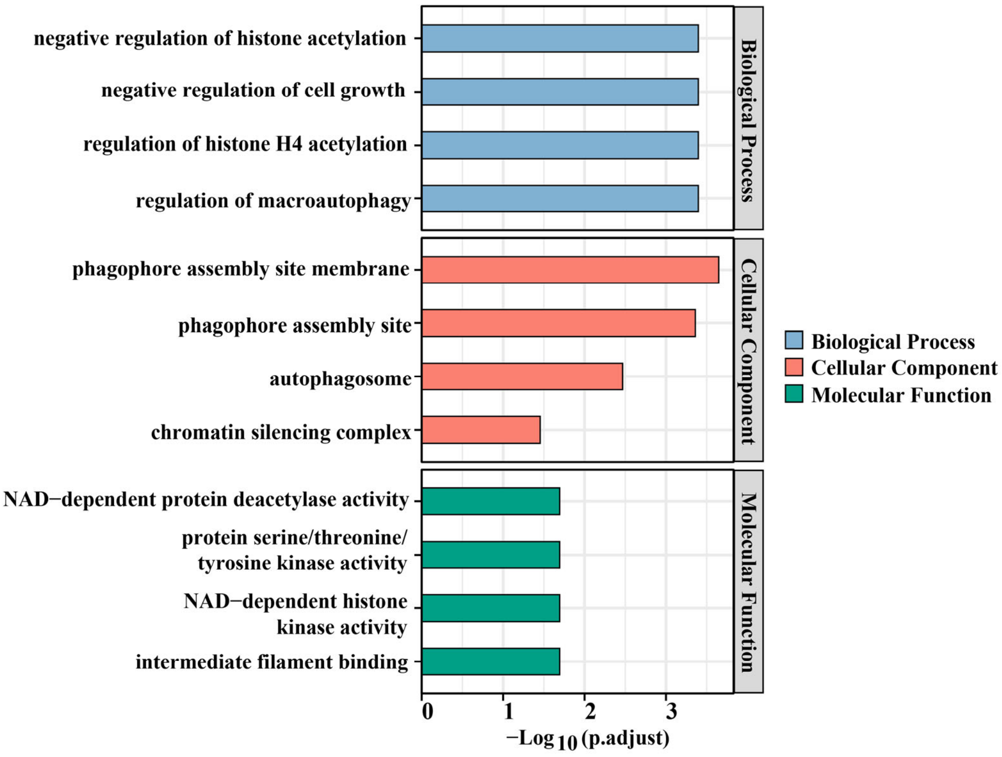

Figure S2. Gene Ontology annotation of miRNA target genes

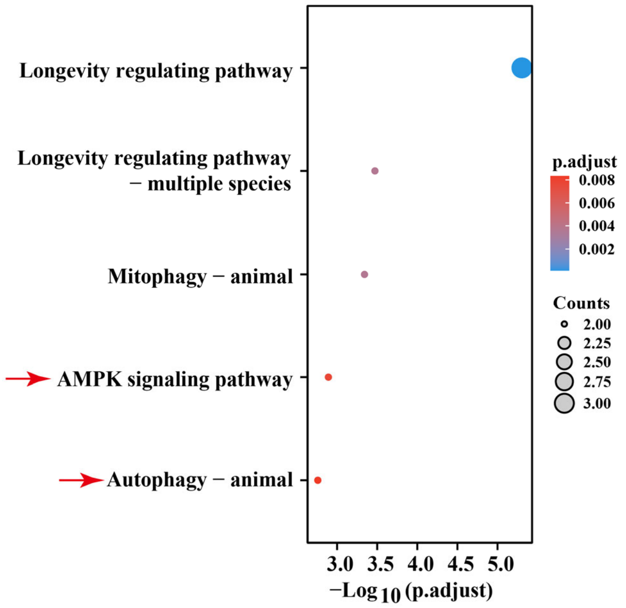

Figure S3. KEGG pathway enrichment analysis of miRNA target genes

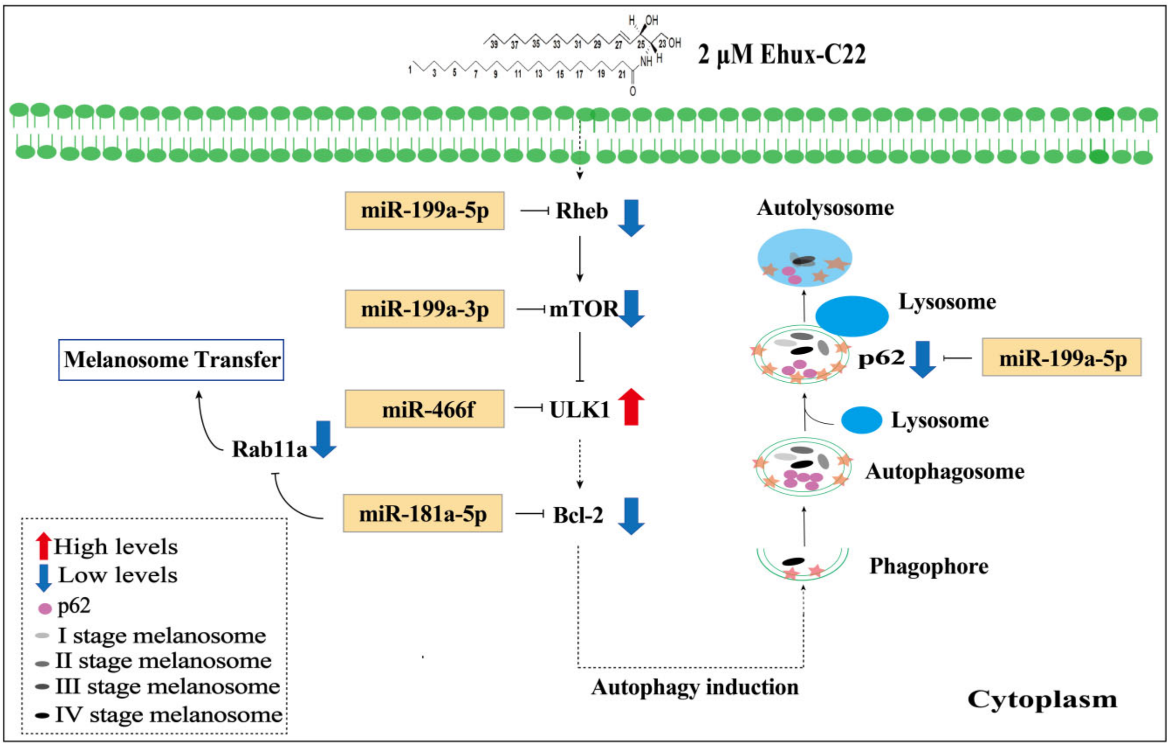

Figure S4. Ehux-C22-induced miRNAs were involved in the regulation of melanosomal autophagy in B16 cells

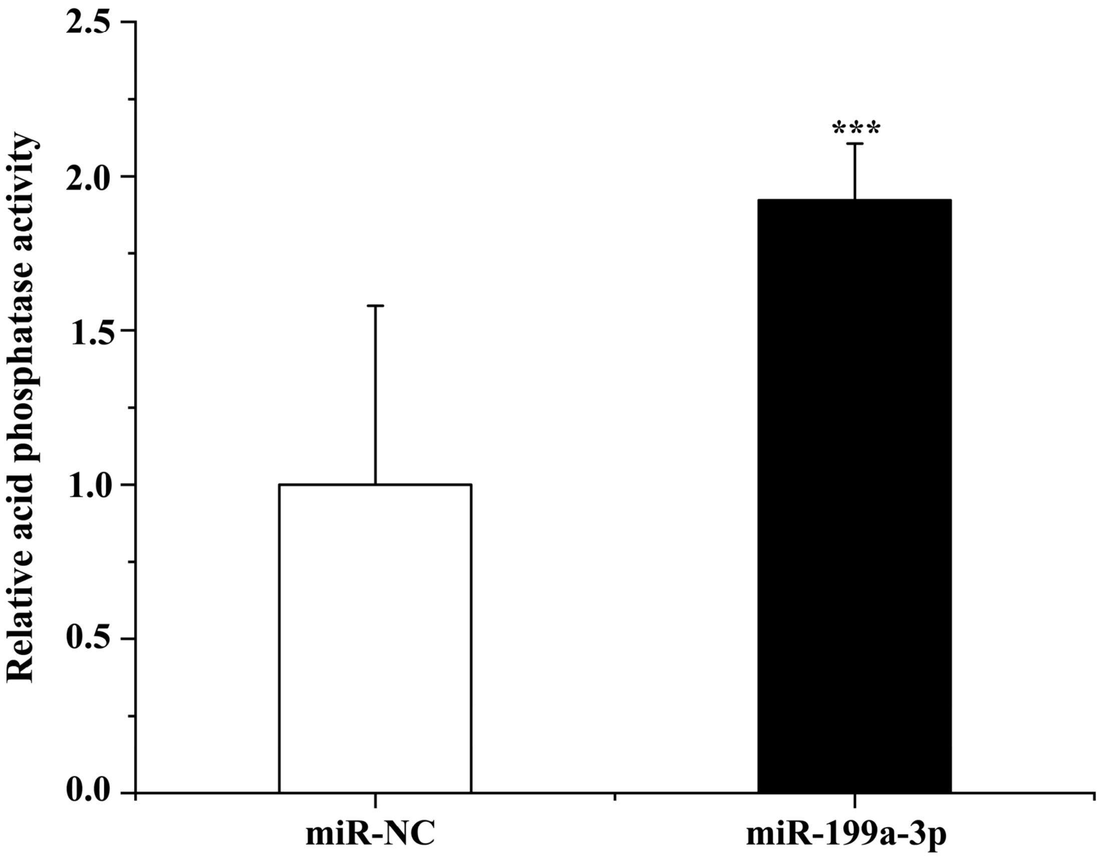

Figure S5. Effect of overexpression of miR-199a-3p on acid phosphatase activity treated with α-MSH in B16 cells.After transfection with miR-199a-3p for 6 h, B16 cells were switched to DMEM high glucose medium containing 0.2 μM α-MSH for 48 h. Western and IP lysates were used to lysate cells, and the supernatant was used to measure the acid phosphatase activity in B16 cells after transfection with miR-199a-3p using 405 nm microplate analyzer. Error bars indicate means ± SD of three biological replicates. Compared with miR-NC group: ***$p < 0.001$.

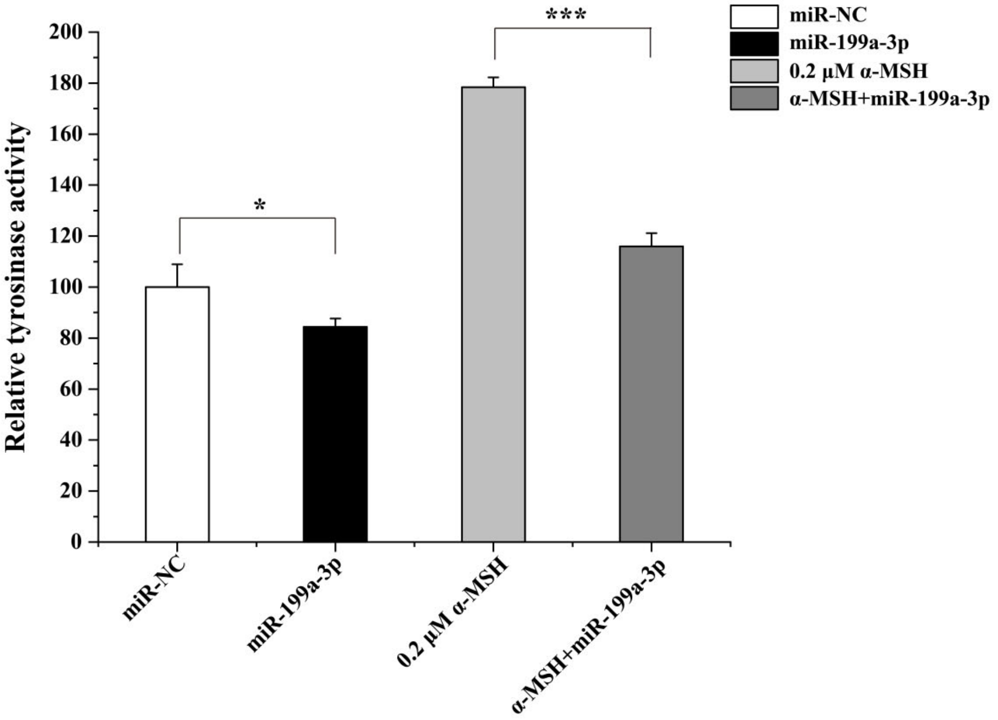

Figure S6. Effect of overexpression of miR-199a-3p on tyrosinase activity after α-MSH-treated in B16 cells. After transfection with miR-199a-3p for 6 h, B16 cells were switched to DMEM high glucose medium containing 0.2 μM α-MSH for 48 h. Tyrosinase activity was measured using L-DOPA oxidation rates. Error bars indicate means ± SD of three biological replicates. Compared with miR-NC or 0.2 μM α-MSH treated groups: $^*p < 0.05$; $^{***}p < 0.001$.

3.Supplementary Tables:

Table S1 Primer sequences used in qRT-PCR of miRNA

miRNA ID	Primer sequences (5'→3')
miR-181a-5p	TGCGAACATTCAACGCTGTCGGTGAGT
miR-199a-5p	GCGCTCCCAGTGTTCAGACTACCTGT
miR-199a-3p	CGTGCGCACAGTAGTCTGCACATTGG
miR-466f	ACGTGTGTGTGCATGTGCATGA
miR-30a-5p	CGCGTGTAAACATCCTCGAC

Table S2. Oligonucleotide primers used for qRT-PCR.

Gene	Primer Orientation	Primer sequences（5’→3’）	Product length /bp
Beclin-1	F	CATCAGAACCTGGGCAACT	184
	R	GGGACTGGAATGGGGACA	
PP2A	F	GATCCTACTACAGTCACTACGC	183
	R	CAGATGCCAGAGATTAATTCGC	
RAB27B	F	GCTGGCTGAAAAATATGGCATA	163
	R	TCCAGAATTTCCTCCATTGACA	
SIRT1	F	CGCTGTGGCAGATTGTTATTAA	171
	R	TTGATCTGAAGTCAGGAATCCC	
Bcl-2	F	GATGACTTCTCTCGTCGCTAC	156
	R	GAACTCAAAGAAGGCCACAATC	
Rheb	F	AAGAAGGACCTGCATATGGAAA	182
	R	GAAGACTTTCCTTGTGAAGCTG	
Epha7	F	CCTGAGGGATTGTAACAGTCTT	164
	R	GGTCACCTTGGGTAAAACTTTC	
Creb1	F	AACTGATTCCCAAAAACGAAGG	185
	R	CACTGCTAGTTTGGTAAATGGG	
Rab11a	F	AGTGATTTACGTCATCTCAGGG	204
	R	GTTGCTTGGAGACATGTCATTT	
LC3I	F	CTTCGCCGACCGCTGTAA	121
	R	TGCGTGGGGTTGAGTTGC	
LC3II	F	CTAACCAAGCCTTCTTCCTCC	197
	R	TGCTGTCCCGAATGTCTCC	
ATG5	F	ATGATTCACGGGATAGAG	184
	R	TGAAGAAAGTTATCTGGGTA	
p62	F	CATCACGCCCTTCACCAGT	142
	R	AGCGGCTTTCCACCAGAGT	
mTOR	F	ACCGTCCGCCTTCACAGATACC	87
	R	GCAGTCCGTTCCTTCTCCTTCTTG	
ULK1	F	TTCAGCACCAGCCGCATTACG	124
	R	CAAAGCCAGCAGAGGGAGCAATC	
MAPK 4	F	CTTCATCAGCACGGAGGACCTTG	116
	R	ACCACTTTGTCACCAACCCTTCTG	
MAPK 6	F	TGCTGACGTAGTTGACAAGTTA	107
	R	TTCCCTTTTCTTGCTTTTCTCG	
β-actin	F	CTCCATCCGGCCTCGCTGT	198
	R	GCTGTCACCTTCACCGTTCC	

Table S3. List of antibodies used in this study.

protein	manufacturers	dilution rate
SQSTM1/p62	Anti-SQSTM1/p62 Rabbit polyclonal antibody (Servicebio, China)	1:1000
LC3I/II	LC3A/B (D3U4C) XP® Rabbit mAb (Cell Signaling technology, USA)	1:1000
phospho-Beclin-1	phospho- Beclin1(Ser15) Antibody (Affinity Biosciences, China)	1:1000
phospho-Bcl-2	phospho- Bcl-2(Ser70) Antibody (Affinity Biosciences, China)	1:1000
ATG5	Rabbit anti-Atg5/Atg5L polyclonal antibody (Bioss, China)	1:1000
mTOR	Rabbit mTOR Monoclonal antibody (Beyotime Biotechnology, China)	1:1000
ULK1	ULK1 (D8H5) Rabbit mAb (Cell Signaling technology, USA)	1:2000
β-actin	Anti-β-actin polyclonal antibody (Beyotime Biotechnology, China)	1:2000
Beclin-1	Anti-Beclin1 Rabbit polyclonal antibody (Servicebio, China)	1:1000
