# Supplementary figures and images for: Ceramide Ehux-C22 Targets the miR-199a-3p/mTOR Signaling Pathway to Regulate Melanosomal Autophagy in Mouse B16 Cells
Source: Int J Mol Sci. 2024 Jul 24;25(15):8061. doi: 10.3390/ijms25158061 (PMC11312279; doi:10.3390/ijms25158061)

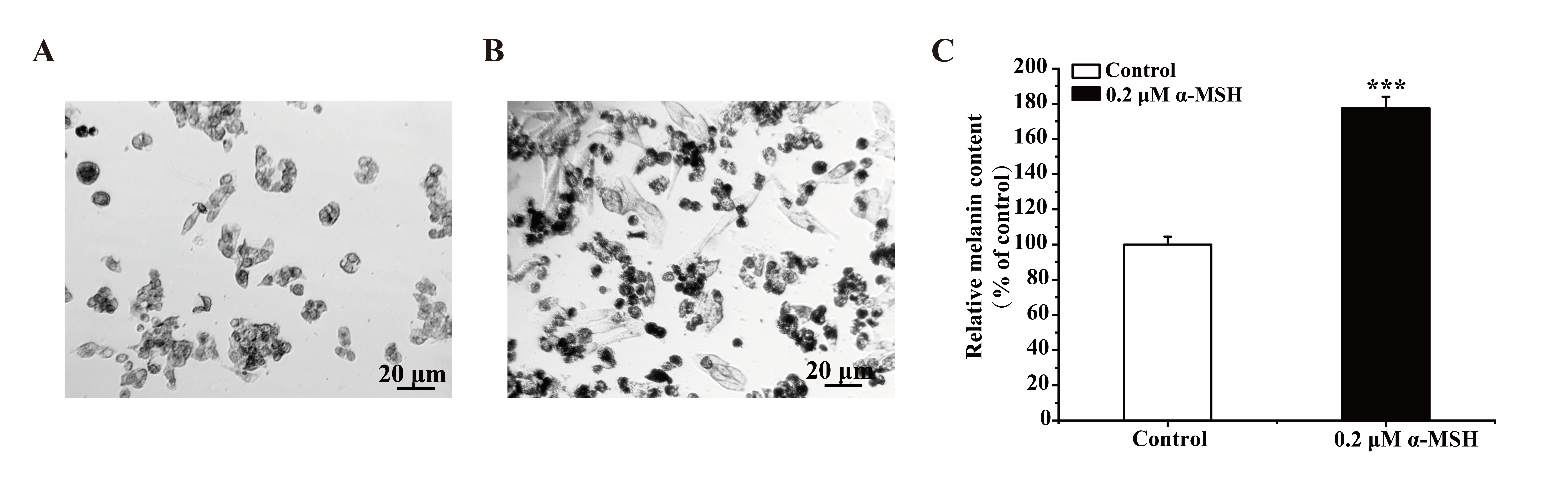

Supplement: Supplementary file 1 [file ijms-25-08061-s001.zip › Figure S1.tif]

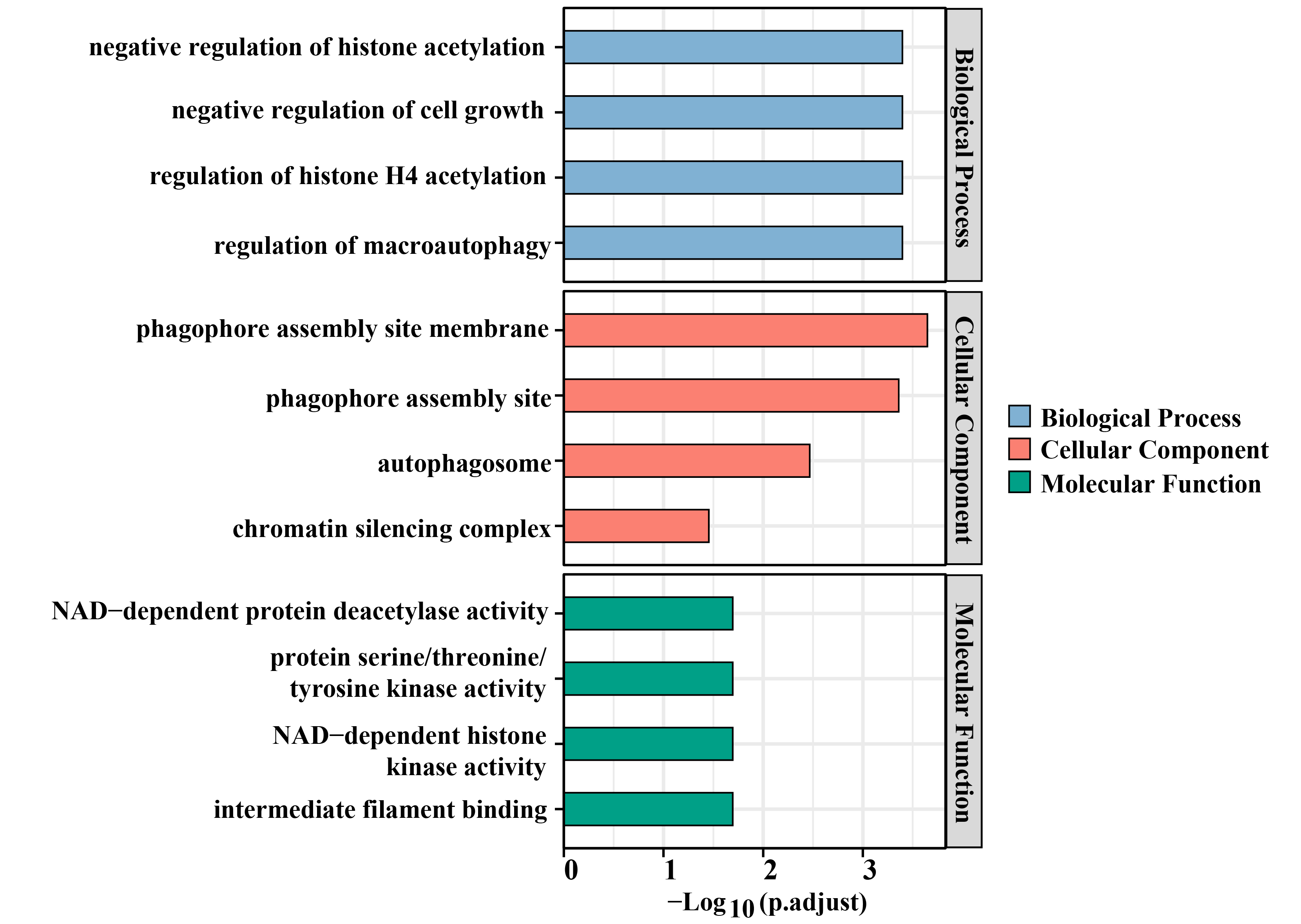

Supplement: Supplementary file 1 [file ijms-25-08061-s001.zip › Figure S2.tif]

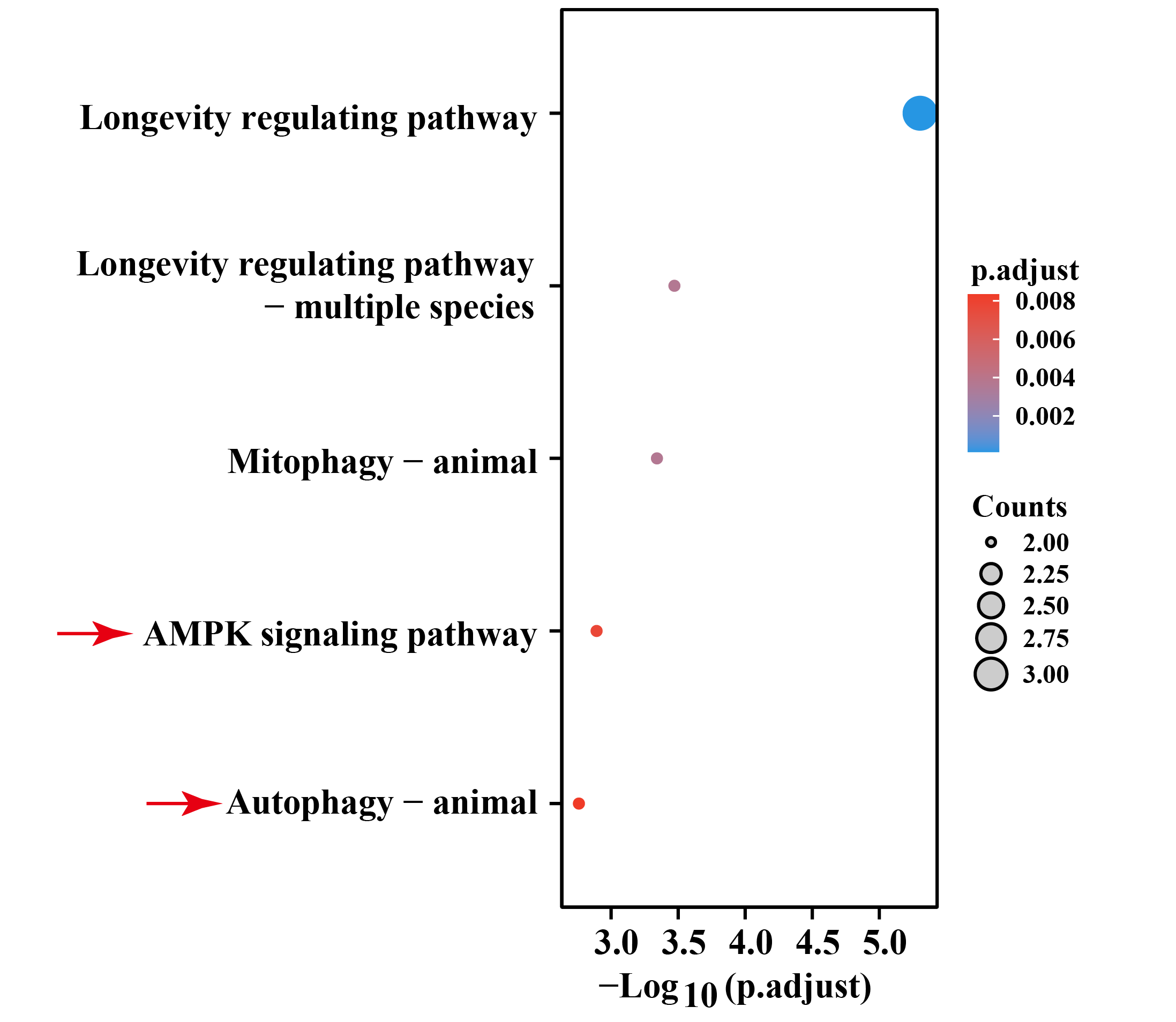

Supplement: Supplementary file 1 [file ijms-25-08061-s001.zip › Figure S3.tif]

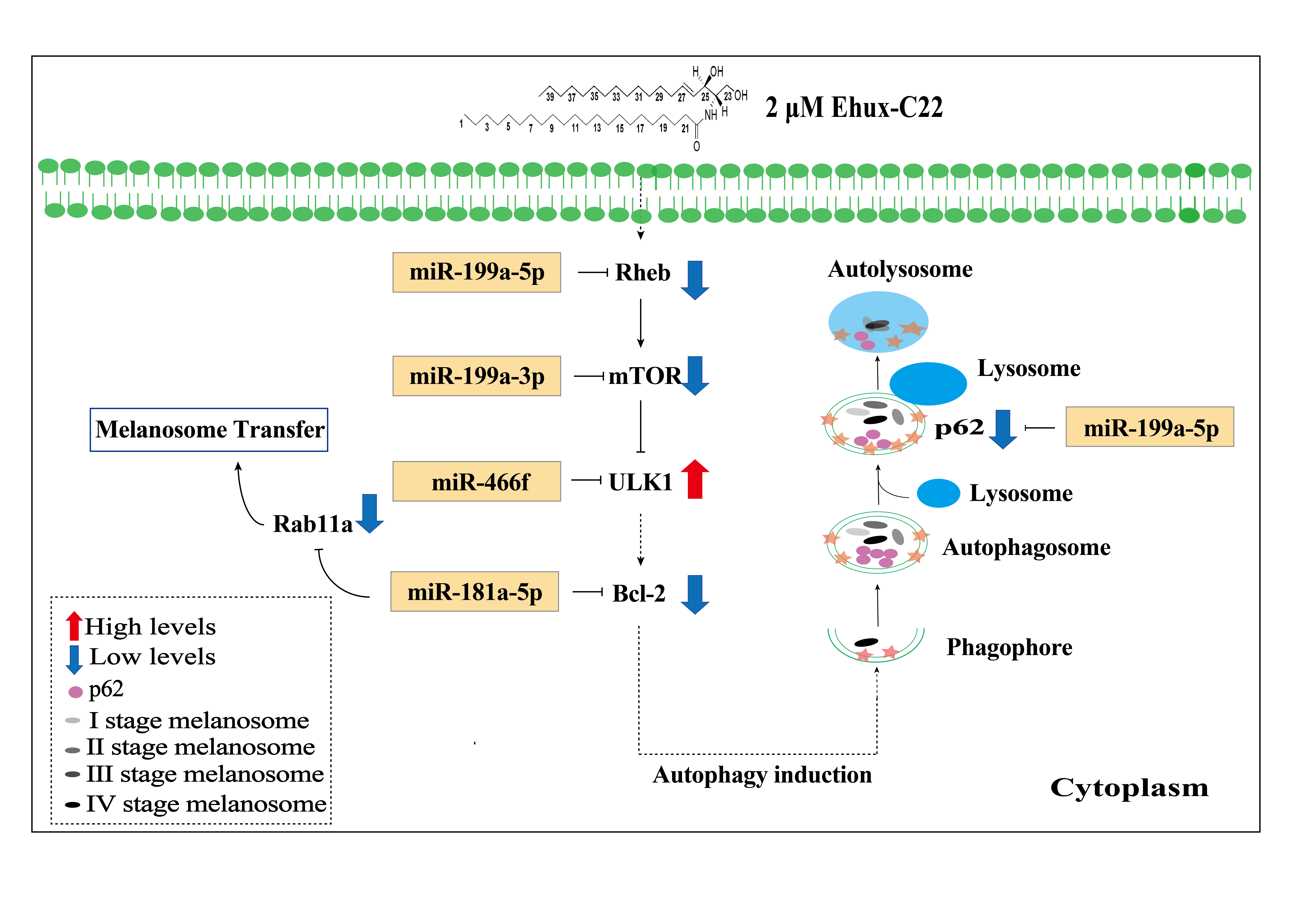

Supplement: Supplementary file 1 [file ijms-25-08061-s001.zip › Figure S4.tif]

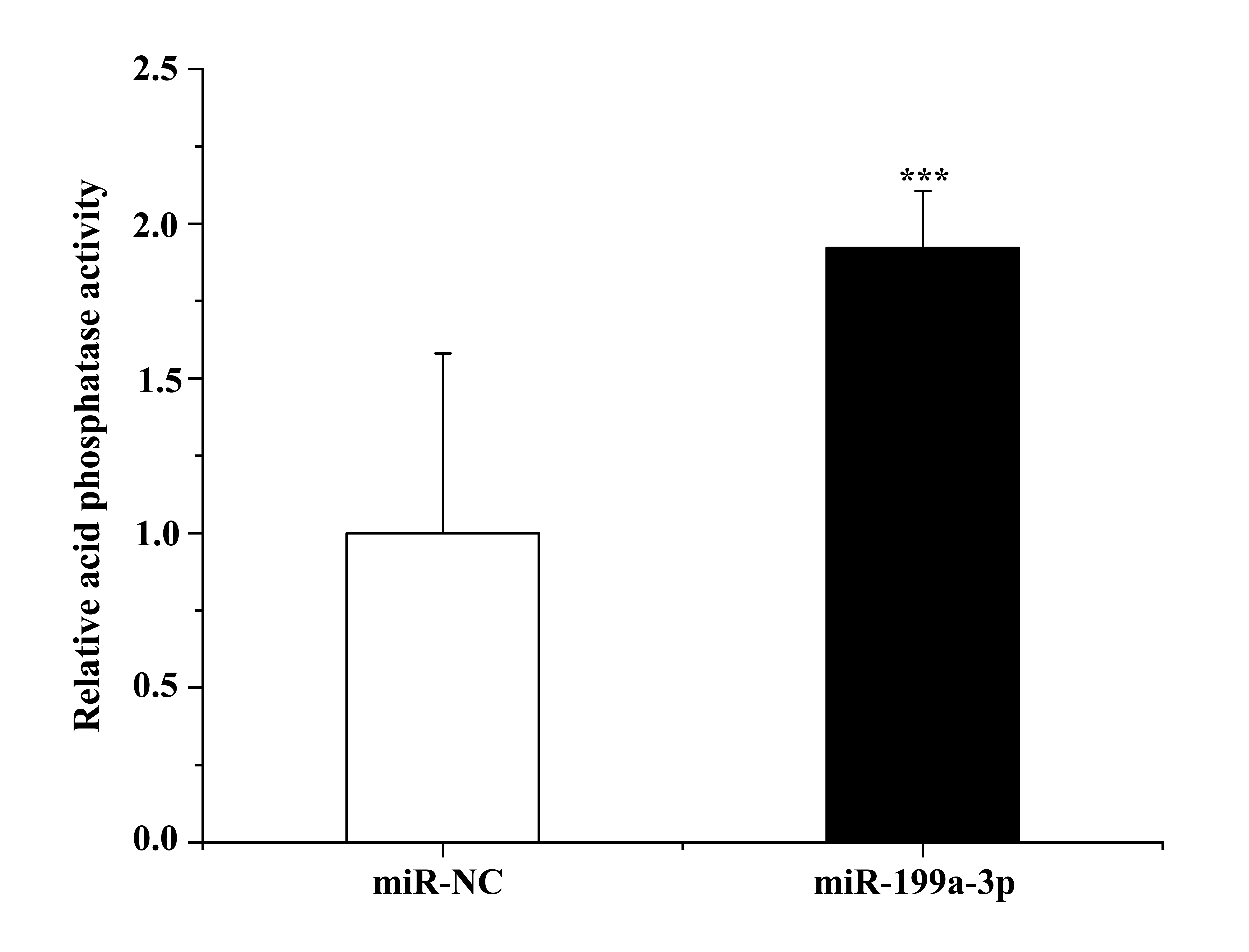

Supplement: Supplementary file 1 [file ijms-25-08061-s001.zip › Figure S5.tif]

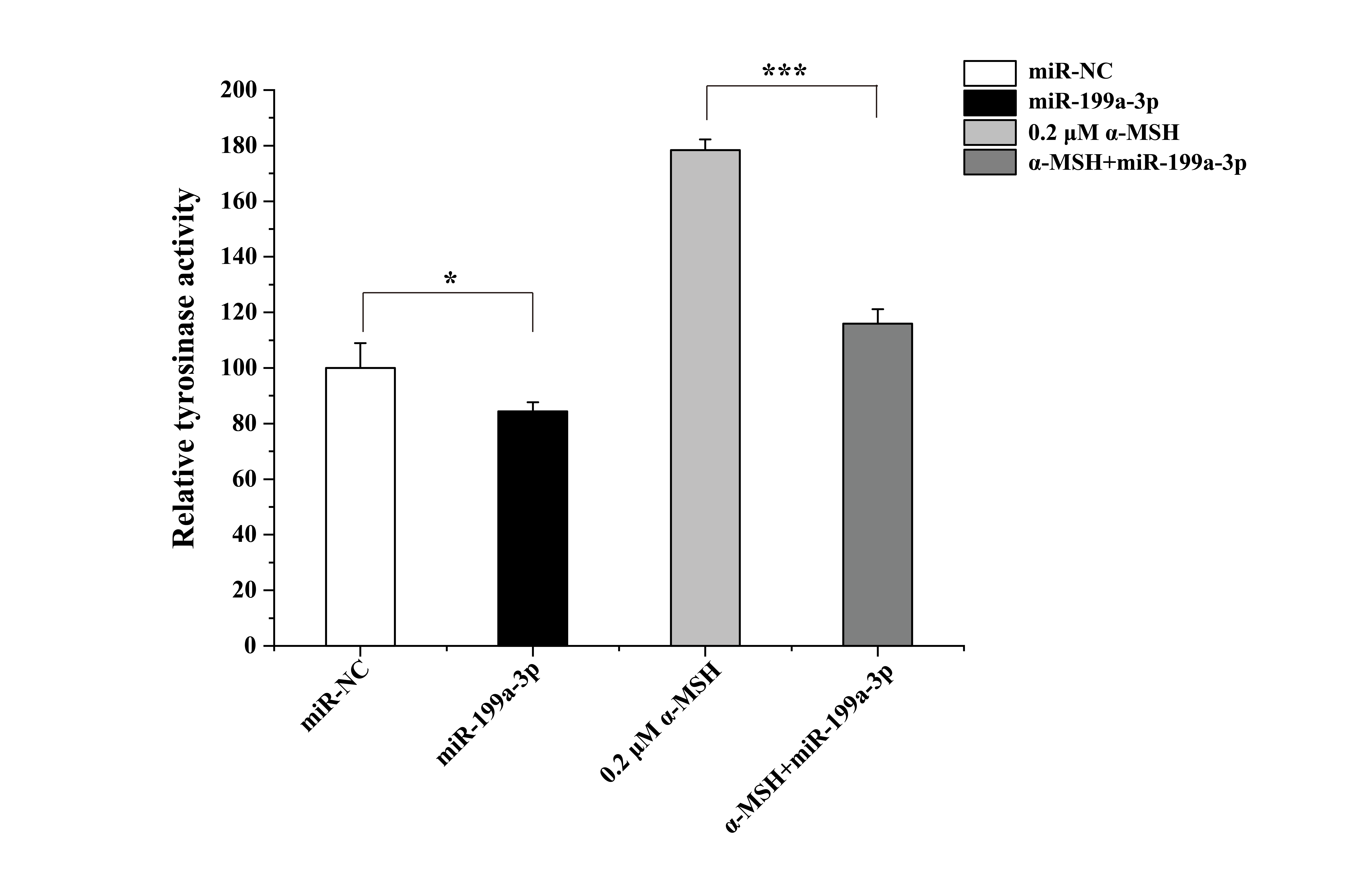

Supplement: Supplementary file 1 [file ijms-25-08061-s001.zip › Figure S6.tif]
